# Onboard Pointing Error Detection and Estimation of Observation Satellite Data Using Extended Kalman Filter

**DOI:** 10.1155/2022/4340897

**Published:** 2022-10-07

**Authors:** R. Dhanalakshmi, N. P. G. Bhavani, S. Srinivasulu Raju, Pundru Chandra Shaker Reddy, Dinesh Mavaluru, Devesh Pratap Singh, Areda Batu

**Affiliations:** ^1^Department of Computer Science and Engineering, KCG College of Technology, Karapakkam, Chennai 600 097, Tamilnadu, India; ^2^Department of Electronics and Communication Engineering, Saveetha School of Engineering, Saveetha Institute of Medical and Technical Sciences, Chennai 602105, Tamilnadu, India; ^3^Department of Electronics and Instrumentation Engineering, VR Siddhartha Engineering College, Vijayawada, Andhra Pradesh, India; ^4^School of Computing and Information Technology, REVA University, Bengaluru, Karnataka, India; ^5^Department of Information Technology, College of Computing and Informatics, Saudi Electronic University, Riyadh, Saudi Arabia; ^6^Department of Computer Science & Engineering, Graphic Era Deemed to Be University, Dehradun 248002, Uttarakhand, India; ^7^Department of Chemical Engineering, College of Biological and Chemical Engineering, Addis Ababa Science and Technology University, Addis Ababa, Ethiopia

## Abstract

The satellite communication is embellished constantly by providing information, ensuring security, and enables the communication among huge at a particular time efficiently. The satellite navigation helps in determining the people's location. Global development, natural disasters, change in climatic conditions, agriculture crop growth, etc., are monitored using satellite observation. Hence, the satellite includes detailed information data, and it must be protected confidentially. The field of the satellite is enhanced at an astonishing pace. Satellite data play an important role in this modern world; hence, the onboard-satellite data must secure through the proper selection of error detection and estimation schema. Lightweight deep learning algorithm based on Extended Kalman Filter (KFK) is proposed to detect and estimate onboard pointing error such as an error in attitude and orbit. The Extended Kalman Filter (EKF) is widely used in the satellite system. EKF is utilized in this proposed model to detect the onboard pointing error such as attitude and orbit determination. An autonomous estimation of orbit position is possible through space-borne gravity. The information obtained through the observation of satellite data is compared with the accurate gravity model in detecting the error. The utilization of EKF reduces the dependence of the ground tracking system in satellite determination. The orbital altitude and orbital position are the most important challenges faced in the satellite determination system. The satellite model using the Extended Kalman Filter is an optimum method in estimating the orbital parameters. The errors in the linearization process are detected, and this can be overcome through the proper selection of linear expansion point with the EKF algorithmic model with the Jacobian matrix calculation. The results show that the EKF implementation helps in attaining better accuracy than other methodologies. Its contribution is enormous to many space missions, autonomous rendezvous and docking for manned and unmanned missions (e.g., ISS operations and beyond, in-orbit servicing, and in-orbit refueling), routine satellite OD operations, orbital debris removal systems, Space Situational Awareness (SSA) operations, and others.

## 1. Introduction

In recent times, the global telecommunication system is more dependent on satellite communication. The satellite is more important in a contemporaneous application such as long-distance cellular calls, radio, and cable television. The satellite design includes the Global Positioning System (GPS) which provides the information regarding the present location and helps in directing us to the place we want to go. Satellite is usually of two types: manmade and natural. Earth and moon are the natural satellites, whereas the manmade satellites are machines that are launched into space and orbits around a body in space. The source error in GPS is atmospheric interference, and the calculation and rounding errors are done using ephemeris data error and multipath effects. The error detection and estimation in the satellite are performed using more algorithmic methods, namely, Extended Kalman Filter (EKF), Unscented Kalman Filter (UKF), and particle filter [1–3]. In the contemporaneous applications, the optimum algorithmic method in error detection and estimation is Extended Kalman Filter (EKF) [4]. The features which make the EKF practically suitable are easy implementation, reduced complexity during computation, and less hardware [5]. The optimum method is EKF which is ideally employed in satellite projects implementation. Alsat-1, SNAP-1, and UoSAT are examples of onboard satellite computers that operate normally [6, 7]. Though EKF has more advantages, it is not an optimal estimator (it is optimal if the measurement and the state transition model are both linear, as in that case the extended Kalman filter is identical to the regular one). It is unable to adjust itself as per the sensor uncertainty.

The algorithmic model is also developed for the accurate determination for the Low Earth Orbit (LEO) [8]. In this algorithmic model, IGS orbits and accurate clock are used for the GPS satellite. The difference obtained between the code-derived position and phase-derived position helps in identifying the position of the satellite. The satellite position and processing speed can be monitored through the positioning of the orbit model in the desired position. The orbit model is fitted depending on the least square adjustment that requires the pseudo-observations. The pseudo-observation is formed by the combination of estimated position from both the code observation and phase observation. Unscented Kalman Filter (UKF) is utilized in the development of an onboard orbit determination algorithm to satisfy the space-borne GPS receiver applications [9]. In onboard processing, accurate orbit navigation is attained through the employment of geopotential, atmospheric drag, the pressure of solar radiation, and the gravity of the Sun and Moon. The propagation of orbit is measured through the theoretical calculation method, namely, the Runge–Kutta method. The orbit motion is estimated through the implementation of the Cowell method. The position of the orbit in an artificial satellite could be determined using the least square algorithm where this algorithmic model includes sequential rotation and GPS receiver's data for the estimation purpose [10]. The Extended Kalman Filter (EKF) and the GPS form an algorithmic model to determine onboard orbit in a satellite. This algorithmic model is simple and compact; hence, its computational cost is very low. The state vector, bias, GPS receiver clock drift rate, position and velocity composition, and drift are determined through the utilization of Extended Kalman Filter (EKF). The onboard error detection and estimation in the satellite are effective in the case of the Extended Kalman Filter (EKF) algorithmic model [11].

## 2. Related Works

The dynamic model can determine the orbit. The system model is more stable and provides accurate information regarding the orbital position [12]. The observation helps in estimating the error and helps in determining the geometrical orbit. This is more challenging as the model requires accuracy in observation; hence, it is a difficult task that traces the accurate orbit position [13]. The association of dynamical state and the information gathered from the geometric observation results in the development of kinematic orbit estimation. This kinematic method based on the observation quality can provide the accurate orbit computation point [14]. The drawbacks mentioned above can be overcome by the proposed reduced dynamic orbit determination. The target can be achieved by the geometric measurement and dynamic force model which consists of sequential filtering [15, 16].

The conventional orbit determination has powerful ground computing with the available ground-based tracking data. Many tracking stations are set up to provide the information from the observed data, and the gathered information is sent to the International GNSS Service (IGS) to develop three different types of orbital products, namely ultra-rapid orbit, rapid orbit, and final orbit. These orbital products can achieve accuracy [17]. When the observation length is small or troposphere delay is severe, then the accuracy of the ground geometric measurement method is poor. The processing system based on the ground-based data is not highly secured. In the case of natural calamities such as water disasters and earthquakes, ground devices are affected [18]. An autonomous satellite navigation system developed in space generates information regarding orbit position and the inter-satellite velocity which is independent of ground-based support.

Autonomous space-based satellite navigation has improved reliability and stability [19]. The accurate orbit determination is achieved through the implementation of satellite navigation as it provides very precise information than the ground-based support [20]. Though the complexity is more in the satellite navigation, the benefits are also simultaneously high. The complex functional features such as fixed the transceivers in the satellite can send and receive signals. The programs are inbuilt in the satellite to perform automatically. High computing potential, high energy, and high reliability are required for the autonomous performance of satellites. If there is no availability of tracking data, then the autonomous navigation faces difficulty in orbit measurement. During the analysis of the orbit parameter, rotational error may result in rank defect if it is not esteemed [21].

The batch processing mode is the aptest conventional satellite navigation system for data smoothing. The orbit estimation process can be performed by collecting a huge number of data, and these data are even suitable for postprocessing. This conventional satellite navigation consists of computing and storage resources to satisfy the space-based environment. The sequential method is far better than the batch processing mode as this method generates the new observation for the accurate orbit determination. This method requires a very low computing capability and less memory. Thus, for the implementation of autonomous satellite navigation, this sequential method is mostly preferred as it requires fewer hardware resources and battery power. The filtering algorithms include the sequence processing which is well known as the Kalman filter.

The recursive Kalman filter is the most effective in determining the internal state of the proposed system. The Kalman filter is more apt for the linear system, whereas the contemporaneous application such as orbit determination comes under the nonlinear satellite navigation systems [22]. The most familiar method in the filtering method is Extended Kalman Filter (EKF). The standardized linear recursive Kalman filter algorithm uses the Taylor series approximation [23]. The estimation of nonlinear state, navigation systems, and GPS are determined by the Extended Kalman Filter (EKF) system model. The orbit determination in the contemporaneous is estimated through EKF. In EKF, during the kinematic calculation, the previously available state estimation is required. The orbit determination and autonomous satellite navigation are independent of the historical observation while increasing computational efficiency. In comparison with the traditional methods, EKF has the potential to predict accurately using the previous states. EKF forecasts orbit determination along with the velocity satellite. Accurate orbit determination, satellite attitude coefficients [24, 25], atmosphere, velocity, and clock are estimated using EKF.

Accuracy in determination can be achieved through the utilization of a reduced dynamic model based on the processor of the satellite. The stability of the filter algorithm is affected due to the error ignorance in the dynamic model. The error in the no-modeling system is compensated using the dynamic noise [26, 27]. Jacobian matrices along with the Extended Kalman Filter (EKF) implementation faced more challenges in some satellite systems. These challenges are solved through the development of the Unscented Kalman Filter (UKF). The big observations error, sample intervals, and large initial errors are overcome using UKF [28, 29]. Some other filter algorithms are H_*∞*_filter and particle filter (PF). The performance speed is high in PF in comparison with EKF and UKF [30].

In the case of satellite orbit determination, the EKF algorithm is the most preferred algorithmic model in the field of satellite [31]. The high-order truncation error is the difficulty faced due to the implementation of the first-order Taylor series in the EKF algorithm. The distinction observed from the processing model and practical model causes the linearization and approximation error. The nonlinear is the important feature in the proposed model. The higher the rate of nonlinear level, the higher will the error in the system model. EKF also depends on the difference between the linear expansion point and the original state. The divergence is caused by incorrect Jacobian matrix computation [32]. These are the consequences which result in filtering error over space [33]. The satellite communication is the latest emerging field and it helps in agriculture, whether monitoring and cultivation etc. there may be certain error that occurs in the space station and transfer wrong information in order to correct that the EKF filter has been used and the way of estimation error filtering is way better than the other filters [34, 35].

## 3. Materials and Methods

### 3.1. System Nonlinear Degree

The system nonlinear degree is the important feature considered in the satellite determination. In the case of the nonlinear system model, the Taylor series expansion is used in the orbit determination. The system consists of many nonlinear characters that correspondingly increase the linearization error. An accurate linear model is achieved only with a zero-linearization error. The satellite orbital position keeps on changing due to the nonlinear degree of force model. The elliptical orbit in a satellite consists of perigee and apogee [36]. At the perigee and apogee points, the nonlinear degree is higher than at the orbital position. The perigee position faces more complexity due to drags. The precession of the orbit is caused by the apogee. Hence, the linearization error is larger at the perigee and apogee points.

### 3.2. Linear Expansion Point

The time of the linear function and predicted time can be acquired under ideal conditions without any error. The orbit determination is not like its original state due to the random errors at the linear expansion point which results in a linearization error in a force model. If the linear expansion error increases, then the linearization error also increases. The linearization faces difficulty in attaining the accuracy of the linear expansion point. The point nearer to the original state must ignore the magnified error in an orbit determination task. Orbit elements, broadcast ephemerides, and geometric approaches are some of the parameters considered in the determination process. The various determination approaches result in different accuracy rates. The satellite traction by ground-based and space-based stations faces challenges in fixing the initial orbital position. The filter approach is unstable when the big bias occurs periodically in the initialization phase. In case of any disaster, the satellite loses its orbital capability.

### 3.3. Jacobian Matrix Calculation

The linearization applies to the nonlinear system only when the Jacobian matrix calculation exists. The orbit determination is very challenging in the force model while executing practically and analytically using the Jacobian matrix. The Jacobian matrix must be implemented very carefully. The Jacobian matrix is implemented in converting the errors of a nonlinear variable space to linearized function space. The Jacobian matrix calculation is depending on the expansion point. The proper selection of expansion points is necessary for meeting the contemporaneous application of satellite. In the case of an improper Jacobian matrix, EKF is leading to an unstable and divergence system. During the filtering procedure, the complex influence is ignored. This algorithmic model has a very good performance evaluation.

### 3.4. Extended Kalman Filter (EKF)¶

The attitude and orbit determination of the satellite are determined through the implementation of Extended Kalman Filter (EKF). In case of gyroscope malfunctions, this filter model is inconsiderable for measurements. The REKF is employed in this proposed system as follows [37]. The seven-dimensional state vector is given as follows:

(1) $$\begin{array}{c} X={\left[q,\omega \right]}^{T}={\left[q_{1},q_{1},q_{1},q_{1},\omega_{x},\omega_{y},\omega_{z}\right]}^{T}\text{.} \end{array}$$

**Step 1 .**

Propagation cycle:

The numerical integration represents the dynamic states of the satellite.

(2) $$\begin{array}{c} {\bar{q}}_{k}={\hat{q}}_{k}+\frac{1}{2}\overset{t_{k+1}}{\underset{t_{k}}{\int }}\Omega \left(\omega_{k}^{{}^{\circ}}\right)q_{k\;}dt\text{,}\\ {\bar{\omega }}_{k}={\hat{\omega }}_{k}+\overset{t_{k+1}}{\underset{t_{k}}{\int }}\left[I_{s}^{-1}\;\left(T_{GG}+T_{D}-T_{C\;}-\omega_{k}\times \left(I_{S\;}\omega_{k}+h\right)\right)\right]\;dt\text{,}\\ {\bar{X}}_{k}=\left[\begin{array}{c} {\bar{q}}_{k}\\ {\bar{\omega }}_{k} \end{array}\right]\text{.} \end{array}$$

The covariance matrix of the predicted error is given as follows:

(3) $$\begin{array}{c} {\bar{P}}_{k}=\phi_{k}{\hat{P}}_{k}\phi_{k}^{T}+Q_{k.} \end{array}$$

Here, the covariance matrix process is denoted as Q_k_ and the state transition matrix is denoted as*ϕ*_k_.

The state transition matrix is expressed as follows:

(4) $$\begin{array}{c} \phi_{k}\approx I_{7\times 7}+\left[\begin{array}{cc} {\left.\frac{\dot{\partial q}}{\partial q}\right|}_{{t=t}_{k}} & {\left.\frac{\dot{\partial q}}{\partial \omega }\right|}_{{t=t}_{k}}\\ {\left.\frac{\dot{\partial \omega }}{\partial q}\right|}_{{t=t}_{k}} & {\left.\frac{\dot{\partial \omega }}{\partial \omega }\right|}_{{t=t}_{k}} \end{array}\right]T_{S}. \end{array}$$

Here, I_7×7_ is the representation of identity matrix with 7 × 7 dimension.

The sampling period is expressed as T_S_=t_k+1_ − t_k_.

**Step 2 .**

Correction cycle:

The observation matrix is estimated as follows [7]:

(5) $$\begin{array}{c} H_{k}=\left[\begin{array}{cc} \frac{\partial {\tilde{B}}_{k}}{\partial \left(q_{1},q_{2},q_{3},q_{4}\right)} & \frac{\partial {\tilde{B}}_{k}}{\partial \left(\omega_{x},\omega_{y},\omega_{z}\right)}\\ \frac{\partial {\tilde{\omega }}_{k}}{\partial \left(q_{1},q_{2},q_{3},q_{4}\right)} & \frac{\partial {\tilde{\omega }}_{k}}{\partial \left(\omega_{x},\omega_{y},\omega_{z}\right)} \end{array}\right]\text{.} \end{array}$$

The Kalman gain K_k_ is tuned by introducing a noise scale factor in the filter which is given as follows:

(6) $$\begin{array}{c} {\bar{s}}_{k}=\frac{e_{k}^{T}e_{k}-tr\left\{H_{k}{\bar{P}}_{k}H_{k}^{T}\right\}}{tr\left\{R\right\}}, \end{array}$$

here, e_k_ is the representation of residual term or innovation sequence.

The residual term is expressed as follows:

(7) $$\begin{array}{c} {\bar{e}}_{k}=\left[\begin{array}{c} {\tilde{B}}_{k}\\ {\tilde{\omega }}_{k} \end{array}\right]-\left[\begin{array}{c} {\hat{B}}_{k}\\ {\hat{\omega }}_{k} \end{array}\right]. \end{array}$$

The magnetometer value is determined with $\hat{B}=A\left(\hat{q}\right)B^{{}^{\circ}}\text{.}$ Here, the trace of the related matrix is represented as tr{∙}.

The Kalman gain is determined through the following expression:

(8) $$\begin{array}{c} K_{k}={{\bar{P}}_{k}H_{k}^{T}\left(H_{k}{\bar{P}}_{k}H_{k}^{T}+{\bar{S}}_{k\;}R\right)}^{-1}\text{.} \end{array}$$

The expression for the estimation of covariance correction matrix is given as follows”

(9) $$\begin{array}{c} {\hat{P}}_{k}=\left(I_{7\times 7}-K_{k}H_{k}\right){\bar{P}}_{k}, \end{array}$$

here, the corrected error covariance matrix is denoted as ${\hat{P}}_{k}$ and the value of *R* gives the noise measurement of the covariance matrix. Thus, the noise measurement of the magnetometer sensors and gyroscope is obtained [38–45].

The expression for the corrected state vector is given as follows:

(10) $$\begin{array}{c} {\hat{X}}_{k}=\left[\begin{array}{c} {\hat{q}}_{k}\\ {\hat{\omega }}_{k} \end{array}\right]={\bar{X}}_{k}+K_{k}{\bar{e}}_{k}. \end{array}$$

**Figure 1:**
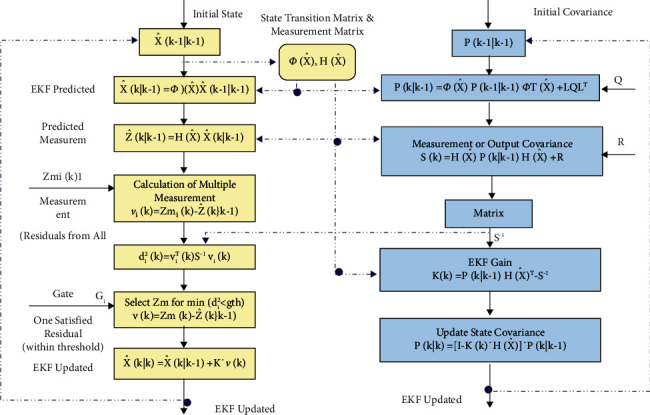
EKF processing flow.

Figure 1 explains the original orbit state vector (i.e., 6 state position and velocity elements) and the 6 × 6 original state error covariance matrix are generally attained from an IOD process, and the entire processing inflow illustrates the typical EKF data processing. The left side of the illustration presents the EKF state processing which consists of two main ways of state prediction via dynamic propagation and state update via sensor measurement processing. The right-hand side of the illustration represents the covariance matrix processing which also consists of two stages: prediction and update. The processing cycle is also repeated with new measures supplied from the detectors, and the prediction step will be propagated via the dynamic process between the measurement times.

## 4. Results and Discussion

The performance of orbit determination is evaluated under the consideration of few features. The main feature important in determination is attaining accuracy. The comparison between the obtained state value and original state value generates the time-domain error curve. This time-domain error curve implies the accuracy of the filter. The consequence faced in the satellite navigation is estimated using the conditional covariance matrix. The accuracy of the prediction is also represented by this matrix.

The variance of the filter is observed and predicted. The information regarding the state components is continuously gathered through the steady observation capability; hence, the occurrence of an error in state condition is estimated through the observation. The features such as sampling rate of data and measurement of noise level are responsible for affecting the steady-state variance. The convergence speed of the filter generates information about the performance of the filter which is inferred during observation.

The Extended Kalman Filter (EKF) is a recursive filter that is effective in the determination of the internal state from noisy measurement series. It only requires an estimation of the previous state for performing its calculation. From the results obtained and the graphs plotted, it is clearly shown that the Extended Kalman Filter with the improvements using the Jacobian matrix calculation point showed fewer truncation errors at a higher order and also increased the orbit accuracy significantly.

**Figure 2:**
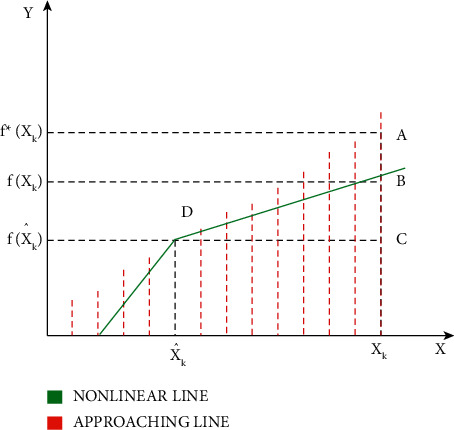
Relationship between nonlinear curve and approaching line.

The relationship between the approaching line and the nonlinear curve is shown in Figure 2. The point of intersection of the approaching line and the nonlinear curve is at point D. The point *D* shows the function of the true state *x'k*. In the true state *xk*, the function of *xk* is given as *f*(*xk*) at point B in the no-linear curve and the function at the same true state is given as *f∗*(*xk*) point A in the approaching line. The difference between the approaching line and the nonlinear curve is given by $\bar{AB}$.

**Figure 3:**
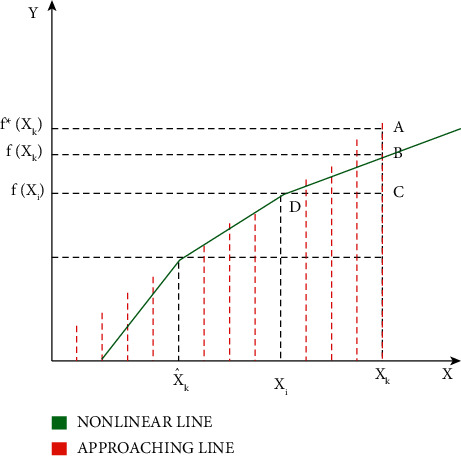
Linearization error when the explosion point is at the estimated interval.

The approaching line in Figure 3 is obtained from the explosion point. The explosion point is placed at the estimation level endpoint. This point is used in obtaining the approaching line. In the Jacobian matrix calculation, the explosion point calculation is randomly chosen from the interval of estimation. Thus, in the true state *xk*, the function of *xk* is given as *f* (*xk*) at point B in the nonlinear curve and the function at the same true state is given as *f∗*(*xk*) point A in the approaching line. The difference between the approaching line and the nonlinear curve is given by $\bar{AB}$.

**Figure 4:**
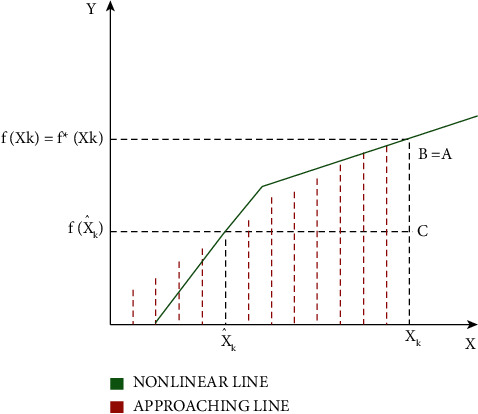
Approaching line is acquired when the explosion point is fixed.

The endpoint of the interval is taken as the explosion points as is shown in Figure 4. The endpoints of the computing interval are used for the Jacobian matrix calculation point. Thus, the explosion points are used in the process of obtaining the approaching line. Thus, in the true state *xk*, the function of *xk* is given as *f* (*xk*) at point B in the nonlinear curve and the function at the same true state is given as *f∗*(*xk*) point A in the approaching line. In the above curve, both the points overlap, and thus the difference between both the points becomes zero. Thus, this makes clear that the approaching line error due to truncation becomes nil.

**Figure 5:**
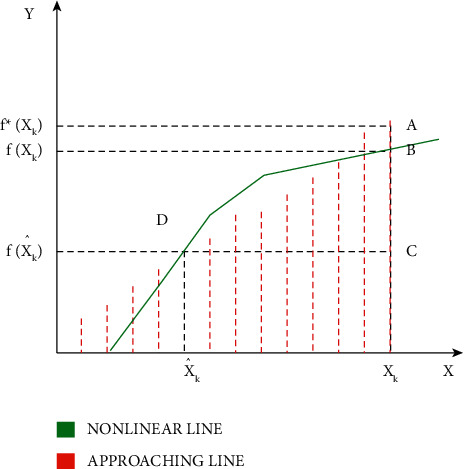
Linearization error at the estimated interval is very small.

Figure 5 includes the estimation interval which is chosen for both the explosion point and for the Jacobian matrix calculation point. In the true state *xk*, the function of *xk* is given as *f* (*xk*) at point B in the nonlinear curve and the function at the same true state is given as *f∗*(*xk*) point A in the approaching line. In the above curve, both the points overlap, and thus the difference between both the points is less. Thus, the error due to the truncation and localization is found to be very low.

## 5. Conclusion

In this proposed system model, onboard pointing error such as an error in attitude and orbit determination is detected and estimated through the development of lightweight deep learning based estimation algorithm on the Extended Kalman Filter (EKF) with the Jacobian matrix calculation. The field of the satellite is enhanced at an astonishing pace as the data available in the satellite are more valuable and sensitive. Thus, there is a demand for error detection, and estimation is essential to protect the satellite data. The satellite determination system is mainly based on the gyroscope partial failure during the estimation. The Extended Kalman Filter (EKF) algorithmic model is examined in predicting the error in the satellite system. The error in the onboard pointing is estimated accurately with the better filtering performance. The Extended Kalman Filter (EKF) is utilized as a backup determination system to protect the microsatellite mission. The nonlinearity, observation noise, and initial condition errors are some of the errors occurring at the linear approximation. The proper selection of linear expansion points enables the performance of the linearization process. The EKF satellite determination system has a higher accuracy rate and strong reliability. This shows that EKF is the optimum method for contemporaneous orbit determination. In the future, Extended Kalman Filter (EKF) will be utilized for the development of autonomous satellite navigation. Hence, the LEO satellite determination is better analyzed using the improvised model [32].

## Data Availability

The datasets used and/or analyzed during the current study are available from the corresponding author on reasonable request.
